# Targeting TRAF6/IRF3 axis to inhibit NF-κB-p65 nuclear translocation enhances the chemosensitivity of 5-FU and reverses the proliferation of gastric cancer

**DOI:** 10.1038/s41419-024-07290-5

**Published:** 2024-12-20

**Authors:** Shitong Chen, Dong Zhang, Yi Du, Junbo Shi, Sikuan Gu, Xujun Zhou, Huijuan Yu, Feng Wang, Jinfei Chen, Hongjuan Cui

**Affiliations:** 1 State Key Laboratory of Resource Insects, Medical Research Institute, Southwest University, Chongqing, 400715 China; 2 Chongqing Engineering and Technology Research Center for Silk Biomaterials and Regenerative Medicine, Chongqing, 400716 China; 3 Department of Oncology, the First Affiliated Hospital of Wenzhou Medical University, Wenzhou, Zhejiang 325035 China

**Keywords:** Cancer, Cell biology

## Abstract

Chemoresistance poses a significant clinical challenge in the treatment of gastric cancer (GC), while its underlying molecular mechanisms are still not fully understood. Post-translational protein modification and abnormal activation of nuclear factor-kappa B (NF-κB) are critical regulators of tumor chemoresistance. This study investigates the role of TNF receptors-associated factors 6 (TRAF6) in 5-Fluorouracil (5-FU) resistant GC. Utilizing short hairpin RNA (shRNA) to suppress TRAF6 expression in 5-FU resistant GC cells across both in vivo and in vitro models, we observed a marked reduction in cell proliferation and tumor growth. Low expression of TRAF6 inhibited nuclear translocation of NF-κB-p65, which was achieved by promoting the expression of Interferon regulatory factor 3 (IRF3). Importantly, TRAF6, an E3 ubiquitin ligase, bound to the IRF3-Δ (SR + IAD) (1-190aa) domain, inducing Lys70 ubiquitination of IRF3 to regulate its protein stability, with ubiquitin K48 residue playing a crucial role in this process. In conclusion, our study reveals the mechanism by which the TRAF6/IRF3 axis decreases GC’s cells sensitivity to 5-FU by promoting nuclear translocation of NF-κB-p65, offering valuable insights into overcoming chemoresistance in GC.

## Introduction

Gastric cancer (GC) is a common malignancy worldwide. According to GLOBOCAN 2022, GC ranks fifth globally in incidence and mortality, with death accounting for nearly 70% of new cases [1]. Currently, surgery has been used as a main treatment for GC at all stages [2]. Chemotherapy is an indispensable treatment for both preoperative and postoperative intervention and for patients with advanced GC whose local cancer is unresectable, recurrent or metastatic, in order to reach the purpose of inhibition of cancer progression [3].

5-fluorouracil (5-FU) is defined as a fluoropyrimidine analogue used to interfere with cancer cell growth. It requires conversion into active metabolites within the body to exert therapeutic effects [4]. As a first-line chemotherapy drug for GC, 5-FU has made great progress in clinical treatment, but the 5-year survival rate of patients remains low. This is mainly due to the presence of congenital or acquired resistance, which hinders the drug’s effectiveness [5]. Presently, the latent mechanism of 5-FU resistance in GC remains unclear, and reducing chemoresistance represents the main challenge to improve overall survival. Therefore, exploring targets at the molecular level is urgently needed to benefit some GC patients.

Studies have shown that tumor chemoresistance is related to ubiquitination modification [6–8]. TNF receptor associated factor 6 (TRAF6) is a RING domain-containing E3 ubiquitin ligase that plays a role in adaptive and innate immunity, as well as autophagy [9]. It is also widely involved in the process of ubiquitination modification. The overexpression of TRAF6 promotes tumor growth and leads to poorly responses to radiotherapy and chemotherapy. For example, TRAF6 induces AKT ubiquitination and accelerates tumor progression in oral cancer [10], and induces K63 ubiquitination of P53 to promote tumor growth and drug resistance [11]. Additionally, activation of TRAF6 plays an important role in the NF-κB signaling pathway [10, 12, 13]. Although increasing studies have highlighted the role of TRAF6 in tumor regulation, the specific ubiquitination mechanism of TRAF6 in chemoresistant tumor cells remains unclear. Interferon regulatory factor 3 (IRF3) is a critical transcription factor for inducing interferon (IFN) and antiviral genes [14, 15]. Its deficiency has been widely reported to induce susceptibility to viruses and antitumor immunity [16, 17]. There are potential interactions between IRF3 and components of the NF-κB signaling pathway [18, 19], which provide an inflammatory tumor microenvironment promoting tumor survival and metastasis [20]. Here, we provide evidence that TRAF6 is aberrantly overexpressed in 5-FU-resistant GC cells, promoting proliferation and reducing chemosensitivity. Mechanistically, TRAF6 binds to the IRF3-Δ (SR + IAD) (1-190aa), and its activation enhances NF-κB-p65 nuclear translocation by downregulating IRF3 expression, with IRF3 Lys70 ubiquitination plays an important role in this process. Therefore, our results indicate the function of the TRAF6/IRF3 axis in 5-FU-resistant GC cells, and TRAF6 is poised to become an attractive therapeutic target to alleviate chemoresistance in GC.

## Materials and methods

### Cell lines, drugs and antibodies

GC cell lines (BGC-823, SGC-7901) and human embryonic kidney cell lines (HEK293FT, 293 T) were obtained from the American Type Culture Collection (ATCC). The human GC cell lines (BGC-823, SGC-7901) were induced to develop resistance to 5-FU using an in vitro method involving a low-concentration gradient increase combined with high-dose intermittent shock, resulting in the 5-FU-resistant GC cell lines (BGC-823-R, SGC-7901-R), which were obtain from Shanghai MEIXUAN. All cell lines tested negative for mycoplasma, as verified by the Myco-Lumi™ Luminescent Mycoplasma Detection Kit (C0298M, Beyotime). Cycloheximide (CHX), MG132, 5-FU were purchased from MCE (Shanghai, China). Antibodies for IRF3, NF-κB-p65, GAPDH, H3, MYC, FLAG, HA, CDK1, and Cyclin A2 were obtained from Proteintech (Wuhan, China), while antibodies for TRAF6, Ki67 were obtained from Cell Signaling Technology (Shanghai, China). All antibodies were used according to the experimental requirements and instructions for use. The antibody codes are shown in Supplementary Table 1.

### Transfection and infection

Small hairpin shRNAs targeting TRAF6 and IRF3, as well as a negative control shRNA (shGFP), were purchased from BGI and cloned into the pLKO.1 vector. Truncation mutant plasmids for IRF3, including IRF3-Δ (SR + IAD) (1-190aa), IRF3-ΔSR (1-384aa) and IRF3-ΔDBD (191-427aa) were purchased from GeneCreate (Wuhan, China). A ubiquitination plasmid containing an HA tag was purchased from Addgene (Beijing, China). TRAF6-PCDH-CMV-MCS-EF1-Puro and IRF3-PCDH-CMV-MCS-EF1-Puro were obtained from YouBio (Changsha, China). Ubiquitin mutant plasmids with an HA tag (K6R, K11R, K27R, K29R, K33R, K48R, K63R) and IRF3 mutant plasmids with a FLAG tag (K29R, K70R, K193R, K313R) were also purchased from YouBio. The constructed plasmid was mixed with three packaging plasmids and transfected into HEK293FT cells. The supernatant was collected after 48 h of culture, and this was the lentivirus. GC and 5-FU-resistant GC cells were infected with polybrene and the collected lentivirus for two consecutive days, followed by puromycin selection to obtain stable cell lines. The shRNA primer sequences are shown in Supplementary Table 2.

### Nuclear and cytosolic extract preparation

The preparation of nuclear and cytosolic proteins from 5-FU-resistant GC cell lines were performed using a commercial Nuclear and Cytoplasmic Protein Extraction Kit (P0027, Beyotime).

### Western blot (WB) assay

Proteins were extracted from cells using RIPA reagent with PMSF, lysed on ice for 30 min, centrifuged for 10 min at 4 °C. The supernatant was collected, and protein concentration was detected and denatured at high temperature. Proteins of different sizes were separated by 10% SDS-PAGE and transferred to PVDF membranes. After transfer, the membranes were blocked with 5% skim milk powder for 120 min, and the corresponding primary antibodies were added. The following day, the secondary antibody of appropriate species was selected and incubated for 2 h. The signal was detected with enhanced chemiluminescence (ECL) reagent (Clinx Science, Shanghai, China) and visualized with a WB detector (Clinx Science, Shanghai, China).

### Quantitative real-time PCR (qRT-PCR)

Cells were collected using TRIzol reagent. Chloroform, isopropyl alcohol, and ethanol were added for total RNA extraction. RNA concentrations were determined and reverse transcribed to cDNA. The qRT-PCR was performed using designed quantitative primers combined with a quantitative kit from Novoprotein (Suzhou, China). The qRT-PCR primer sequences are shown in Supplementary Table 3.

### Cell proliferation assay

Each well of a 96-well plate was filled with 200 μl of medium mixed with 1000 cells. The experiment was repeated three times for each group. The cell viability was determined by MTT test and the growth curve was drawn based on the absorbance value detected by enzyme standard instrument (Synergy HTX, BioTek, USA).

### EdU staining

The cells were cultured overnight in 24-well plates containing 20,000 cells per well. The next day, the medium was removed and EdU was added to culture for 2 h, followed by fixation for 15 min and aeration for 15 min. After washing, the Click reaction mixture was incubated with the sample at room temperature and away from light for 30 min. Stain nucleus with 1×Hoechst33342. Refer to BeyoClick™ EdU Cell Proliferation Kit with Alexa Fluor 594 (C0078S, Beyotime) for detailed steps. Collect images by fluorescence microscope (Nikon 80i, Tokyo, Japan).

### Plate clone formation assay

Six well plate was prepared with 1000 cells in 3 ml of medium per well. The cell status was monitored regularly and the medium was changed as needed during the culture period. Two weeks later, images were collected using scanner (Perfection V700 Photo, Epson, Japan).

### Soft agar clone formation assay

A 1.2% low melting point agarose solution was prepared and maintained at 55 °C in a water bath. This solution was added to a six-well plate at a ratio of 1:1 with medium, with 1 ml added to each well. This created a 0.6% agarose solution in the wells, which was allowed to solidify at room temperature. 0.3% agarose solution was then prepared, and 1000 cells were thoroughly mixed into it. This cells-agarose mixture was added to 6-well plates. Cells were incubated under thermostatic condition for 4 weeks, and single cell clones were imaged using a white light microscope. Finally, MTT solution was added and incubated in the dark for 30 min, after which images were collected using a scanner (Perfection V700 Photo, Epson, Japan).

### Flow cytometry

Cells were collected and held at 4 °C in 75% ethanol for static fixation for 48 h. After fixation and washing, the staining solution was prepared per the cell cycle kit instructions and incubated in the dark for 20 min. Cells were analyzed by flow cytometry (Sparrow 2040, Cellula, USA). FlowJo software was used for data analysis.

### Co-immunoprecipitation (Co-IP)

The collected adherent cells were lysed on ice with IP lysate added with PMSF for 60 min, centrifuged for 10 min (12,000 g, 4 °C), and the supernatant was collected. Portion of the supernatant were designated as the INPUT group, IgG group, and IP group. The corresponding primary antibody was added to the IP and IgG group. The IgG and IP groups were incubated on a rotating table overnight. The next day, protein A/G agarose beads were added to the samples and incubated for 4 h. After washing five times the samples were denatured and subjected to WB experimental tests.

### Proximity ligation assay (PLA)

Approximately 20,000 cells were added to each of the 24-well plates containing cell slides, and transient transfection was performed. Cells were fixed, permeabilized, blocked, and incubated with primary antibodies after 48 h as previous described [21]. The next day, the cell slides were incubated with PLA probes at 37 °C for 60 min and then with the ligase mixture at 37 °C for 30 min. Finally, the slides were incubated with an amplification enzyme mixture at 37 °C for 100 min (#DUO92101, Sigma-Aldrich). Cytoskeleton and nucleus staining were performed, and signals were captured using a confocal microscope (FluoView FV1000, Olympus, Japan).

### Immunofluorescence assay (IF)

In simple terms, IF followed the same experimental steps as PLA on day one. The cells were incubated the following day with fluorescent secondary antibodies corresponding to the species. The nucleus was then stained using Hoechst 33342 (1:2 000), and signals were captured by confocal microscope (FluoView FV1000, Olympus, Japan).

### Immunohistochemical (IHC) staining

The tumor tissue was embedded, sectioned, dewaxed, dehydrated followed by blocking with goat serum. TRAF6, IRF3, and Ki67 antibodies were diluted and added to the sections, then incubated at 4 °C overnight. The secondary antibody linked to horseradish peroxidase was added the next day and incubated for 20 min, then DAB staining was performed. Finally, the sections were counterstained with hematoxylin, differentiated with hydrochloric acid, dehydrated, etc. The slides were then photographed under the microscope.

### Subcutaneous xenograft assay in vivo

Animal experiments were approved by the Institutional Animal Care and Use Committee of Southwest University (IACUC-20230927-01). All experiments were performed in accordance with the Guidelines for Animal Health and Use (Ministry of Science and Technology of China, 2006). Four-week-old female BALB/C-nu mice were purchased from Cavens Biogle(Suzhou) Model Animal Research Co.Ltd. They were housed in SPF animal houses and randomly assigned into three groups. GC chemoresistant cells statically transfected with shGFP, shTRAF6 or shTRAF6/shIRF3 were injected into one axilla. The weight and tumor volume of the mice were regularly recorded after injection completion, and about 4 weeks later, mice from same batch were euthanized. The subsequent experiments were conducted by randomization and single blind method.

### Database analysis

Patient datasets were obtained from the GEPIA (http://gepia.cancer-pku.cn/detail.php?clicktag=correlation, 2023 Jan 1) and Kaplan-Meier Plotter databases (https://kmplot.com/analysis/index.php?p=service&cancer=gastric, 2023 Jan 1, 2023 May 23). Predicted TRAF6 binding proteins were obtained from the SRTING database (https://cn.string-db.org/, 2023 May 23).

### Statistical analysis

All experimental results from at least three independent experiments. Image J was used to analyze WB band intensities. The statistical analyses were performed using GraphPad Prism 9.5 and Microsoft Excel for Windows 10. Quantitative data are expressed as the mean ± SD. The *P*-values were calculated using two-tailed Student’s t-tests. *P* < 0.05 was considered statistically significant, *p < 0.05, **p < 0.01, ***p < 0.001.

## Results

### High expression of TRAF6 enhances 5-FU resistance in GC cells

**Fig. 1:**
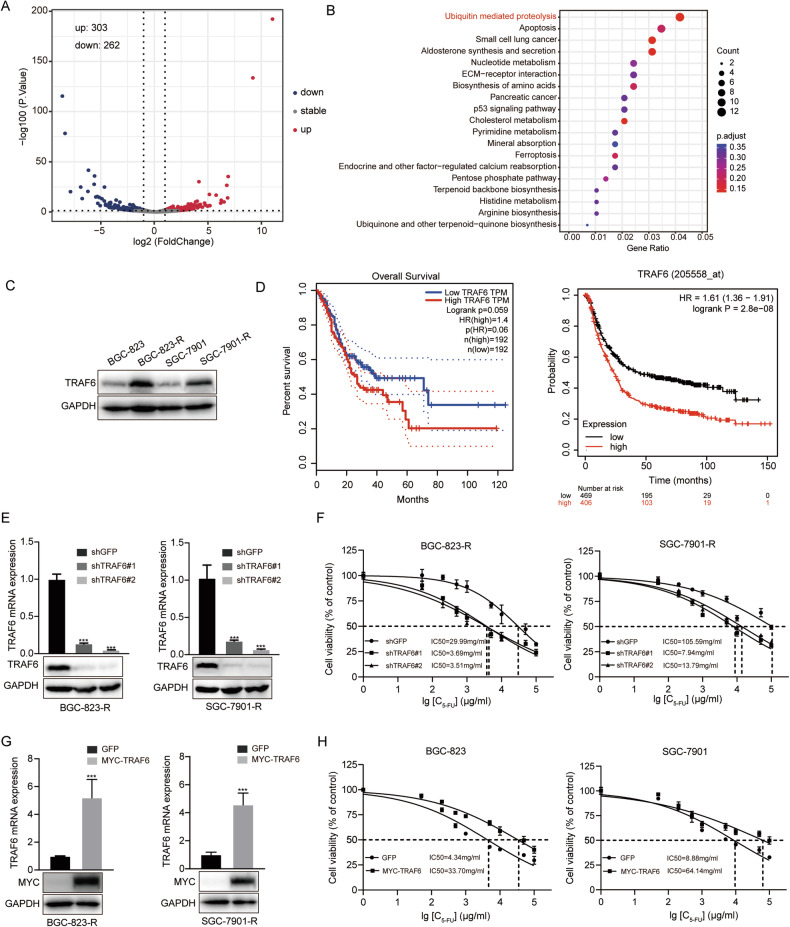
High expression of TRAF6 enhances 5-FU resistance in GC cells. **A** Proteome sequencing analysis was conducted on chemoresistant cells (SGC-7901-R) and chemosensitive cells (SGC-7901), with a visual volcano plot illustrating the number of up and downregulated genes. **B** KEGG pathway enrichment analysis of differential genes in SGC-7901 and SGC-7901-R cells. **C** Western blot was performed to detect the expression of TRAF6 in chemotherapy resistant and sensitive cells. **D** Kaplan–Meier analysis of overall survival probability using GEPIA and Kaplan-Meier (KM) plotter database. **E** Western blot and qRT-PCR assays were conducted to detect the expression levels of TRAF6 in the control and TRAF6-knockdown BGC-823-R and SGC-7901-R cells. Short hairpin (sh) GFP was used as the negative control. **F** The IC50 of 5-FU was measured in TRAF6-knockdown BGC-823-R and SGC-7901-R cells. **G** Western blot and qRT-PCR assays were adopted to detect the expression levels of TRAF6 in the control and TRAF6-overexpressing BGC-823 and SGC-7901 cells. **H** The IC50 of 5-FU was measured in TRAF6-overexpressing BGC-823 and SGC-7901 cells. Data were expressed as the mean ± SD, *n* = 3. Student’s t-test was performed to analyze significance. *P < 0.05, **P < 0.01, ***P < 0.001.

To screen the key genes contributing to 5-FU resistance in GC, we conducted proteomic sequencing analyses of 5-FU-resistant (BGC-823-R, SGC-7901-R) and sensitive GC cells (BGC-823, SGC-7901), in which differentially expressed genes were represented by volcano plot (Fig. 1A). The differentially expressed genes were enriched in the ubiquitin mediated proteolysis pathway by KEGG enrichment analysis, and the expression of ubiquitin ligase TRAF6 in this pathway exhibited particularly prominent differences (Fig. 1B). First, we verified the high expression of TRAF6 in 5-FU-resistant GC cells (Fig. 1C, Supplementary Fig. 1A), then used GEPIA and Kaplan-Meier (KM) Plotter databases to detect positive correlation between aberrant TRAF6 activation and poor prognosis in GC patients (Fig. 1D). Therefore, TRAF6 was selected for further study. Lentivirus-mediated RNA interference was used to knockdown the expression of TRAF6 (shGFP was negative control), successfully downregulating TRAF6 levels in BGC-823-R and SGC-7901-R cells (Fig. 1E). Subsequently, we examined TRAF6 expression effects on GC cell resistance to 5-FU. Results showed that TRAF6 knockdown significantly enhanced 5-FU sensitivity in BGC-823-R and SGC-7901-R cells (Fig. 1F), while overexpression of TRAF6 in BGC-823 and SGC-7901 cells decreased sensitivity (Fig. 1G, H). Overall, TRAF6 is critical for 5-FU resistance in GC.

### TRAF6 depletion inhibits the proliferation of 5-FU resistant GC cells and blocks their cell cycle progression

**Fig. 2:**
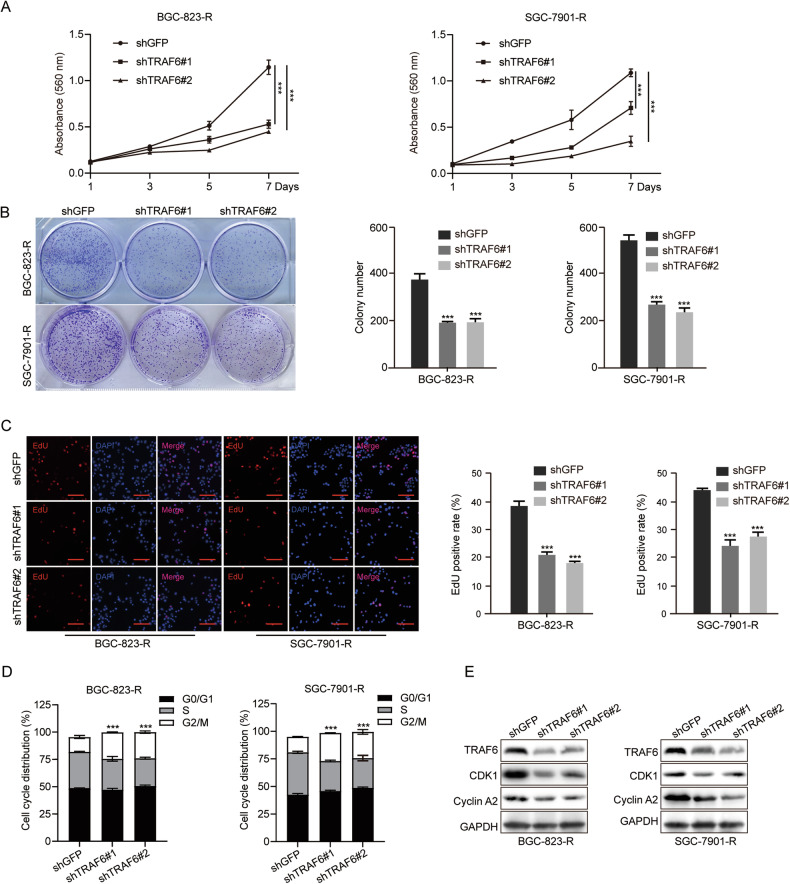
TRAF6 depletion inhibits the proliferation of 5-FU resistant GC cells and blocks their cell cycle progression. **A**, **B** MTT and plate cloning assays were performed to examine the proliferation of the control and TRAF6-knockdown BGC-823-R and SGC-7901-R cells. **C** EdU staining assay was performed to detect the amount of DNA synthesis. Scale bar = 50 μm. **D**, **E** The cell cycle distribution was analyzed in BGC-823-R cells and SGC-7901-R cells by flow cytometry, and G2/M cell cycle-related proteins were detected by western blot assays. Data were expressed as the mean ± SD, *n* = 3. Student’s t-test was performed to analyze significance. *P < 0.05, **P < 0.01, ***P < 0.001.

To study the effect of TRAF6 on 5-FU-resistant GC cells, MTT assays, plate cloning, EdU tests, and flow cytometry were conducted on control and TRAF6 knockdown cells. The results demonstrated that TRAF6 knockdown significantly impacted the proliferation of both BGC-823-R and SGC-7901-R cells. The cloning ability of 5-FU-resistant GC cells was significantly inhibited after TRAF6 knockdown (Fig. 2A, B). EdU tests also showed knocking down TRAF6 dramatically decreased the EdU-positive rate versus controls (Fig. 2C). Flow cytometry revealed the cell cycle of TRAF6-knockdown cells was mainly blocked in the G2/M phase. Consistently, the expression levels of related cyclins CDK1 and Cyclin A2 decreased after TRAF6-knockdown (Fig. 2D, E). These results suggest TRAF6 is crucial for 5-FU-resistant GC cells proliferation.

### TRAF6 promotes the cloning of 5-FU-resistant GC cells in vitro and tumor growth in vivo

**Fig. 3:**
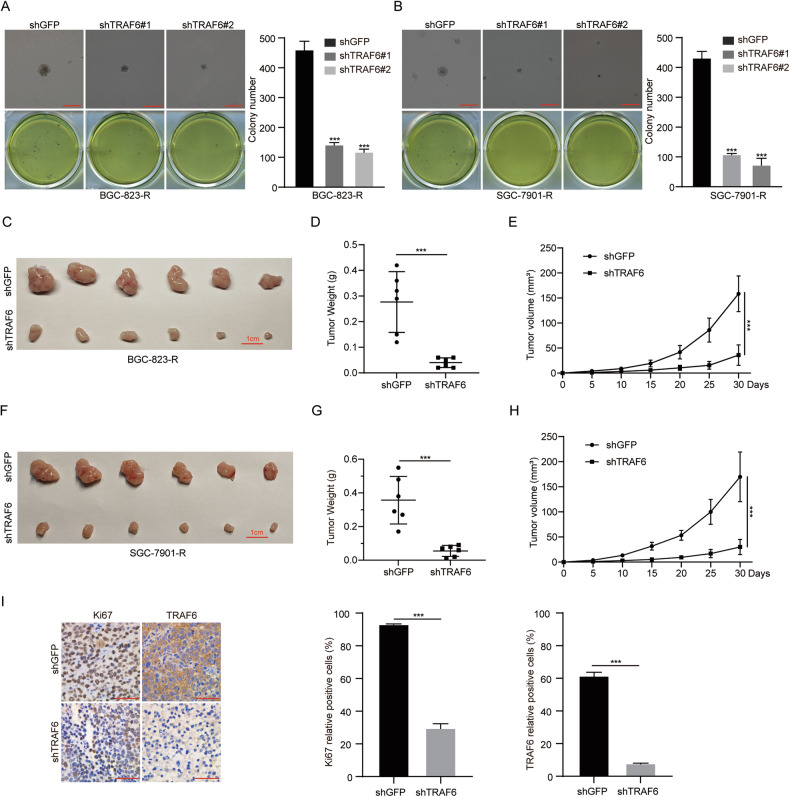
TRAF6 promotes the cloning of 5-FU-resistant GC cells in vitro and tumor growth in vivo. **A**, **B** Soft agar assay was performed to detect the colony formation ability with TRAF6-knockdown cells. The colony quantification results are presented. Scale bar = 200 μm. **C**–**H** Photographs, growth monitoring and weights of the indicated xenograft tumors. Scale bar =1 cm, *n* = 6. **I** Representative IHC images of the positive expression levels of Ki67 and TRAF6. Scale bar = 50 μm. Quantification of TRAF6 and Ki67 expression. Data were expressed as the mean ± SD, *n* = 3. Student’s t-test was used to analyze significance. *P < 0.05, **P < 0.01, ***P < 0.001.

Next, in order to detect the effect of TRAF6 on the tumorigenesis of 5-FU-resistant GC cells, we tested the tumorigenicity of 5-FU-resistant GC cells expressing shGFP or shTRAF6 in vitro by soft agar assay. Results showed fewer and smaller colonies in the experimental group versus control (Fig. 3A, B). This finding was further supported by in vivo tumorigenesis experiments where TRAF6 knockdown BGC-823-R and SGC-7901-R cells were injected subcutaneously into immunodeficient mice. After 30 days, compared with the control group, the TRAF6 knockdown group had slower subcutaneous tumor growth, smaller volume and weight (Fig. 3C–H). We explored if TRAF6 drives tumor progression through promoting proliferation of 5-FU-resistant GC cells. Immunohistochemical staining on the removed mouse tumors revealed significantly lower expression of TRAF6 and Ki67 in tumors with TRAF6 knockdown (Fig. 3I), which clearly proved that TRAF6 promoted the tumorigenesis of 5-FU-resistant GC cells.

### TRAF6 interacts with the IRF3-Δ (SR + IAD) (1-190aa) domain

**Fig. 4:**
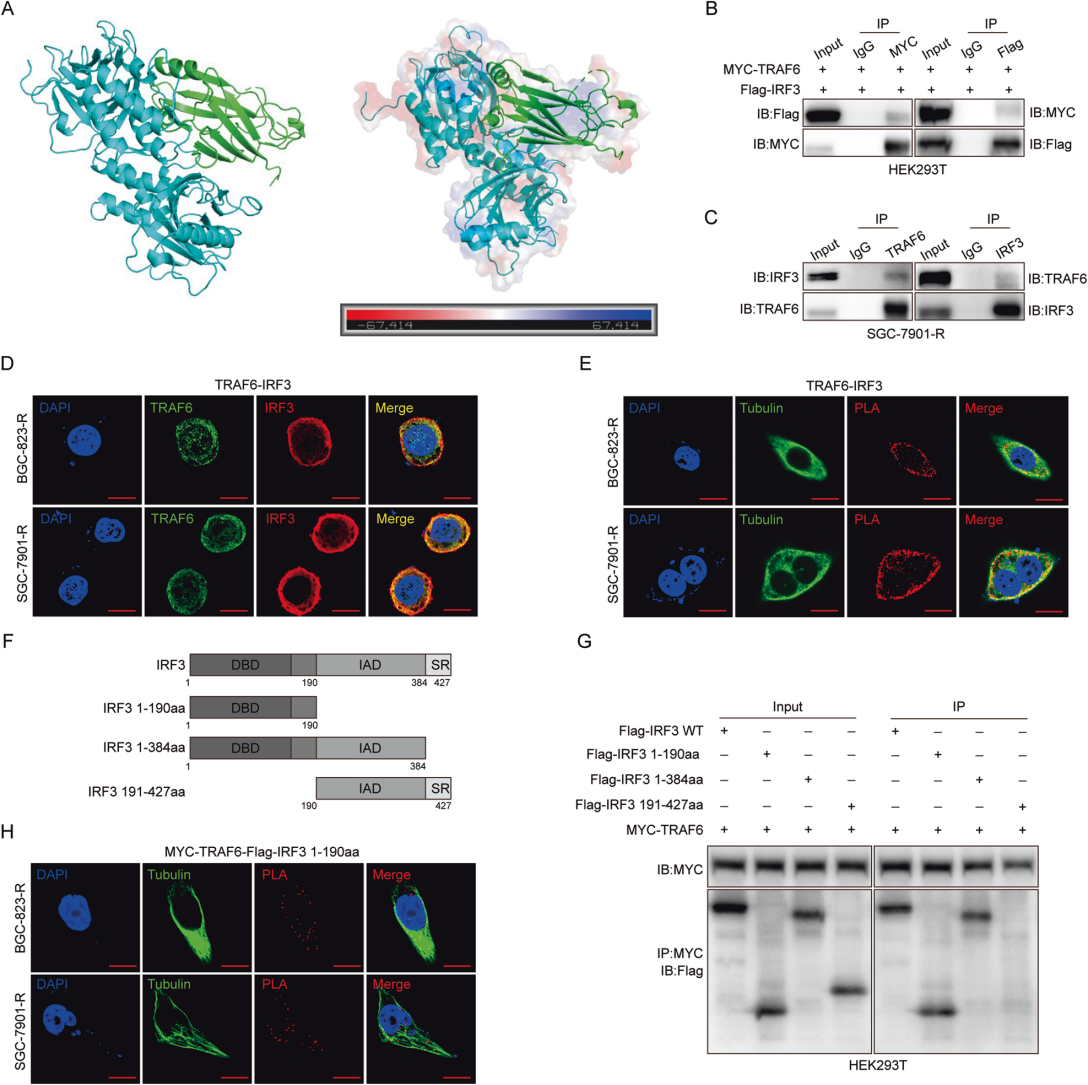
TRAF6 interacts with the IRF3-Δ (SR + IAD) (1-190aa) domain. **A** Download the protein structures of TRAF6 (1LB4) and IRF3 (1QWT) from PDB. Water molecules and receptor proteins were removed using PyMOL, followed by molecular docking. **B**, **C** Co-IP assays were performed to detect interactions between TRAF6 and IRF3 proteins in 293 T and SGC-7901-R cells. **D** Immunofluorescence staining was performed for intracellular localization of TRAF6 and IRF3. Scale bar = 2 μm. **E** The proximity ligation (PLA) assay was performed to detect the interactions of TRAF6 and IRF3 with red indicating a positive signal for PLA. Scale bar=2 μm. **F** Schematic representation of IRF3 and its truncated mutants. **G** Co-IP assays were performed to detect interactions of MYC-TRAF6 and Flag-IRF3 or its truncated mutants. **H** PLA was performed to detect interactions of MYC-TRAF6 and Flag-IRF3(1-190aa) with red indicating a positive signal for PLA. Scale bar = 2 μm.

Subsequently, we discussed the role of TRAF6 in 5-FU-resistant GC cells. Hou et al. suggested that there may be a potential regulatory interaction between TRAFs and IRF3 in the process of antiviral innate immunity [22]. PyMol visual molecular docking showed high binding compatibility for TRAF6-IRF3 under natural conditions (Fig. 4A). The STRING and KM plotter databases also predicted IRF3 as a TRAF6 binding protein, with abnormally activated IRF3 expression negatively correlating with poor prognosis of GC patients (Supplementary Fig. 2A, B). We analyzed the connection between TRAF6 and IRF3 via Co-IP to confirm this finding. Results showed whether exogenous MYC-labeled TRAF6 protein or FLAG-labeled IRF3 protein was immunoprecipitated with FIag or MYC antibody in 293 T, or endogenous TRAF6 or IRF3 protein was immunoprecipitated with IRF3 or TRAF6 antibody in GC cell lines, TRAF6 and IRF3 were bound (Fig. 4B, C). Additionally, immunofluorescence (IF) and proximity ligation assay (PLA) results showed that IRF3 was bound to cytoplasmic TRAF6 in almost all cases (Fig. 4D, E). Human IRF3 protein has three conserved domains consisting of 427 amino acids. To explore which domain of IRF3 binds to TRAF6, we constructed various IRF3 truncates: IRF3-Δ (SR + IAD) (1-190aa), IRF3-ΔSR (1-384aa), IRF3-ΔDBD (191-427) (Fig. 4F). Co-IP results showed that Flag-IRF3 1-190 and Flag-IRF3 1-384 bound to TRAF6, while Flag-IRF3 191-427 did not. It was proved that only Flag-IRF3-1-190 binds to TRAF6 (Fig. 4G). Similarly, this result was also confirmed by PLA experiment (Fig. 4H). In summary, the interaction domain between TRAF6 and IRF3 is IRF3-Δ (SR + IAD) (1-190aa).

### TRAF6 promotes NF-κB-p65 nuclear translocation by inhibiting IRF3

**Fig. 5:**
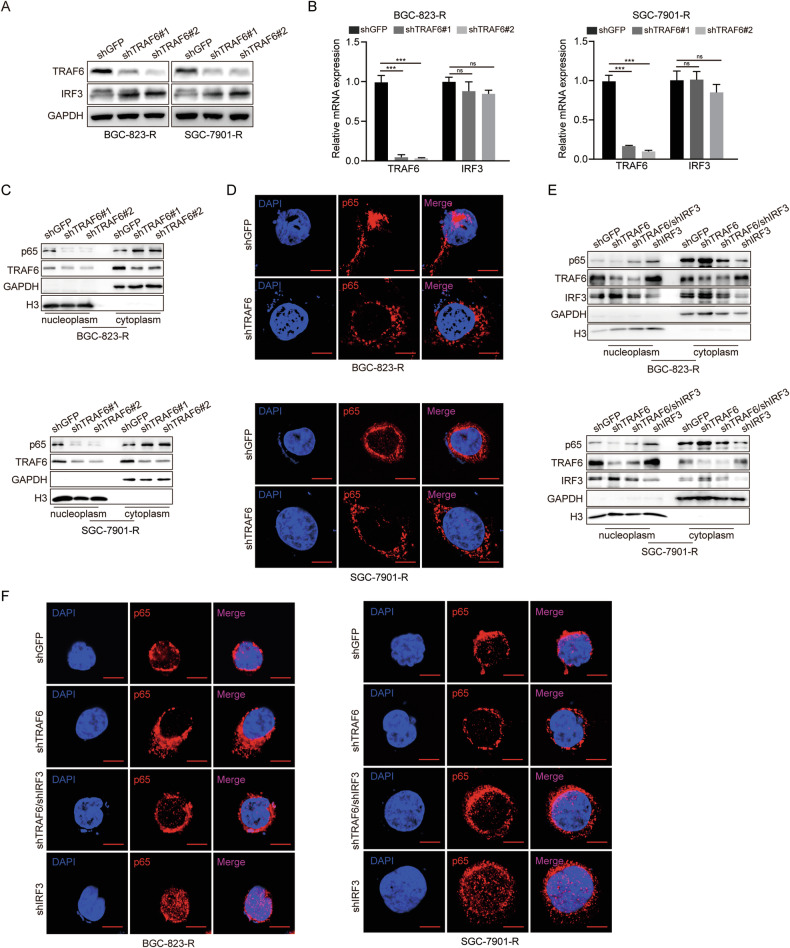
TRAF6 promotes NF-κB-p65 nuclear translocation by inhibiting IRF3. **A**, **B** Western blot and qRT-PCR assays were perform to detect the protein and mRNA levels of IRF3 in TRAF6-knockdown BGC-823-R and SGC-7901-R cells. **C** Western blot analysis was performed to detect the expression level of p65 in TRAF6-knockdown BGC-823-R and SGC-7901-R cells. **D** Immunofluorescence staining was performed for intracellular localization of p65. Scale bar = 2 μm. **E** The Western blot analysis was performed to detect the expression level of p65 after IRF3 knockdown in TRAF6-knockdown cells. **F** Immunofluorescence staining was performed for intracellular localization of p65. Scale bar = 2 μm. Data were expressed as the mean ± SD, *n* = 3. Student’s t-test was performed to analyze significance. *P < 0.05, **P < 0.01, ***P < 0.001.

In order to understand the regulatory relationship of TRAF6 on IRF3, we conducted WB experiments and observed that protein level of IRF3 increased following TRAF6 knockdown, while IRF3 mRNA level remain significantly unchanged (Fig. 5A, B). Given that previous studies have shown that NF-κB signaling pathway is crucial in tumor growth and drug resistance, we further explored the effect of the TRAF6/IRF3 axis on NF-κB-p65 in 5-FU-resistant GC cells, a key player in the NF-κB signaling pathway. Notably, TRAF6 deficiency did not alter the expression level of NF-κB-p65 (Supplementary Fig. 3A). However, nuclear-cytoplasmic separation experiments showed that the nuclear translocation of NF-κB-p65 decreased after knocking down TRAF6 (Fig. 5C), confirmed by immunofluorescence assays (Fig. 5D). Additionally, overexpression of TRAF6 in BGC-823-R and SGC-7901-R cells increased NF-κB-p65 nuclear translocation (Supplementary Fig. 3B), indicating high expression of TRAF6 promotes NF-κB-p65 nuclear translocation in 5-FU-resistant GC cells. Considering previous research results, we hypothesized that TRAF6 may promote NF-κB-p65 nuclear translocation via suppressing IRF3, so we simultaneously performed nuclear-cytoplasmic separation and IF experiments in wild-type, shTRAF6, shTRAF6/shIRF3, and shIRF3 5-FU-resistant GC cells. As expected, IRF3 knockdown rescued the reduction of NF-κB-p65 into the nuclear after TRAF6 knockdown (Fig. 5E, F), so our results show TRAF6 promotes the nuclear translocation of NF-κB-p65 via suppressing IRF3, thereby reducing 5-FU sensitivity in GC cells.

### Lys70 is a major lysine residue of IRF3 for ubiquitination induced by TRAF6

**Fig. 6:**
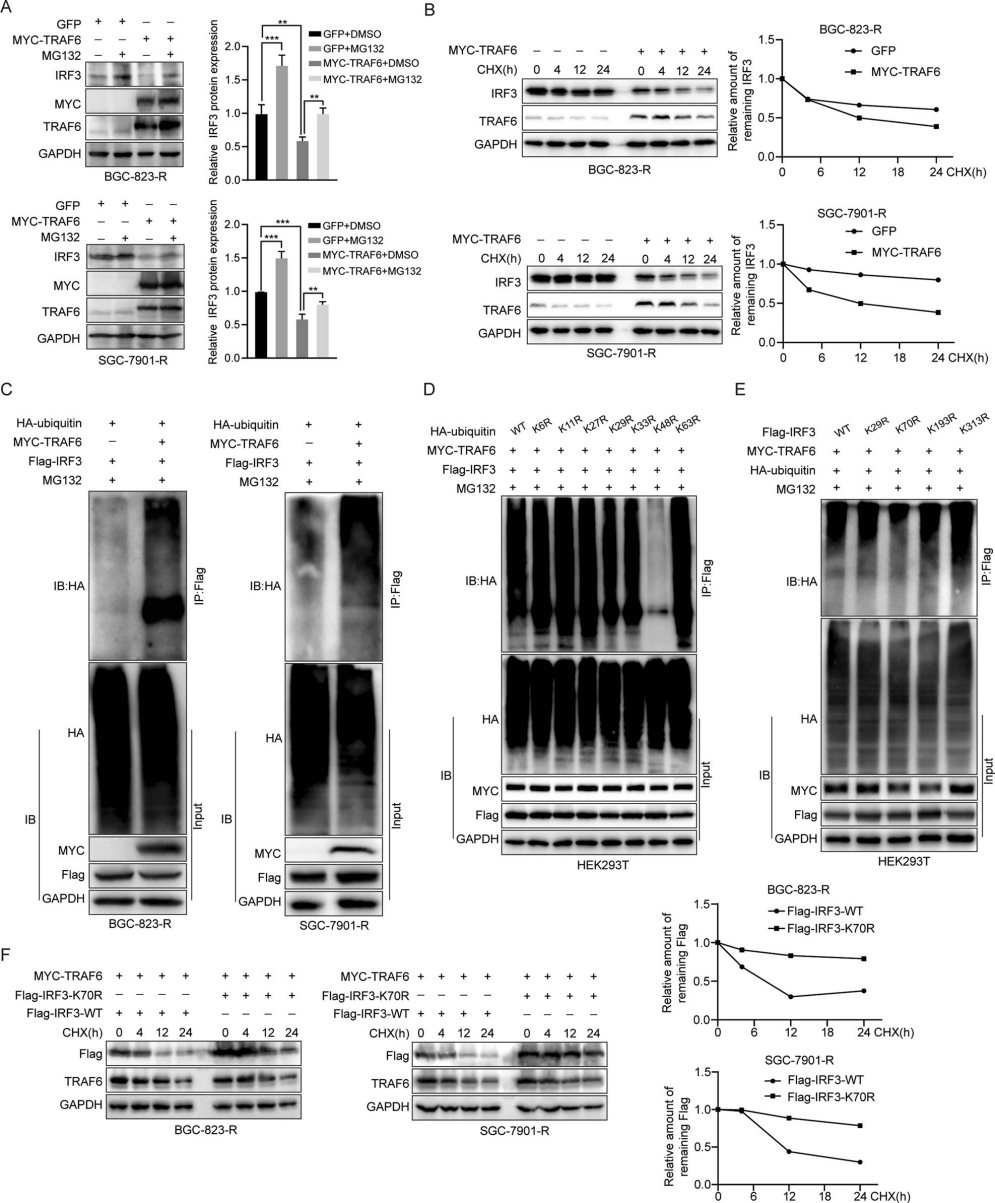
Lys70 is a major lysine residue of IRF3 for ubiquitination induced by TRAF6. **A** Western blot analysis of control and TRAF6-overexpressing BGC-823-R and SGC-7901-R cells treated with MG132 (10 μM) for 6 h. **B** Western blot analysis of control and TRAF6-overexpressing BGC-823-R and SGC-7901-R cells treated with CHX (20 μM) for the indicated times (0 h, 4 h, 12 h, 24 h), Semi-quantitative analysis of IRF3 protein levels was performed using Image J. **C** In the presence of MG132, with or without MYC-TRAF6, Flag-IRF3, and HA-UB plasmids were co-transfected into BGC-823-R and SGC-7901-R cells for ubiquitination assays. **D** In the presence of MG132, MYC-TRAF6, Flag-IRF3, HA-UB, and ubiquitin mutant plasmids (only one lysine residue was mutated to an arginine residue) were co-transfected into 293 T cells for ubiquitination assays. **E** In the presence of MG132, MYC-TRAF6, Flag-IRF3 (wild-type and single-point mutants K29R, K70R, K193R, K313R) and HA-UB were co-transfected into 293 T cells for ubiquitination assays. **F** Western blot analysis of wild-type and IRF3 single-point mutant K70R BGC-823-R and SGC-7901-R cells treated with CHX (20 μM) for the indicated times (0 h, 4 h, 12 h, 24 h), Semi-quantitative analysis of IRF3 protein levels was performed using Image J. Data were expressed as the mean ± SD, *n* = 3. Student’s t-test was performed to analyze significance. *P < 0.05, **P < 0.01, ***P < 0.001.

TRAF6 is widely involved in the process of ubiquitination. Chen et al. found that IRF3 can be degraded as a ubiquitin substrate [23]. To better understand the mechanism of TRAF6 regulating IRF3, we investigated whether TRAF6 regulates the ubiquitination level of IRF3. Proteasome inhibitor (MG132) was added to BGC-823-R and SGC-7901-R cells overexpressing TRAF6. WB analysis showed IRF3 expression significantly decreased after TRAF6 overexpression; however, IRF3 protein expression recovered after MG132 treatment (Fig. 6A). No obvious IRF3 protein recovery was observed after NH4Cl treatment (Supplementary Fig. 4A), indicating that TRAF6 regulates the degradation of IRF3 protein through ubiquitin proteasome pathway. Then cycloheximide (CHX) was added to BGC-823-R and SGC-7901-R cells overexpressing TRAF6 across a time gradient (0 h, 4 h, 12 h, 24 h). The turnover of IRF3 was detected by WB experiments. It was found that the turnover of IRF3 decreased after TRAF6 overexpression, indicating that TRAF6 can regulate the stability of IRF3 (Fig. 6B). To determine how TRAF6 regulating IRF3 stability, we examined its effect on IRF3 ubiquitination in 293 T cells. Co-IP experiments demonstrated IRF3 ubiquitination increased with TRAF6 overexpression in concentration dependent (Supplementary Fig. 4B). Similar results were obtained in BGC-823-R and SGC-7901-R cells (Fig. 6C). In contrast, the downregulation of TRAF6 reduced the level of IRF3 ubiquitination (Supplementary Fig. 4C). Thus, TRAF6 is an E3 ligase of IRF3 and regulates IRF3 degradation via ubiquitination. In order to further identify key lysine residues of TRAF6 regulating IRF3 ubiquitination, we mutated lysine residues at positions 6, 11, 27, 29, 33, 48 and 63 in the HA-UB expression plasmid to arginine and co-transfected these or HA-labeled wild-type plasmids with MYC-TRAF6 and Flag-IRF3 into 293 T cells. The results showed that TRAF6 mediated IRF3 ubiquitination via K48 (Fig. 6D). Since IRF3 was involved in TRAF6-mediated ubiquitination and its key amino acid residues needed to be explored. According to the prediction of GPS-Uber Web Server and Chen et al.[23], we mutated four lysine residues of IRF3, including Lys29, Lys70, Lys193 and Lys313. Mutants Flag-IRF3-K29R, Flag-IRF3-K193R, Flag-IRF3-K313R were strongly downregulated by TRAF6, similar to Flag-IRF3-WT. However, Flag-IRF3-K70R mutation largely eliminated the TRAF6-induced IRF3 degradation, suggesting Lys70 as the main ubiquitin receptor of IRF3 targeted by TRAF6 (Supplementary Fig. 4D). Consistently, K70 mutation significantly inhibited TRAF6-mediated ubiquitination of IRF3 (Fig. 6E). Finally, analysis of protein stability of K70-mutated IRF3 under TRAF6 regulation showed a slower degradation rate compared to wild-type (Fig. 6F). In summary, TRAF6 as an E3 ligase induces ubiquitination of IRF3 at Lys70, regulating the stability of IRF3 protein. Ubiquitin K48 also plays a key role.

### Depletion of IRF3 promotes the proliferation and tumor growth in 5-FU-resistant GC cells with TRAF6 knockdown

**Fig. 7:**
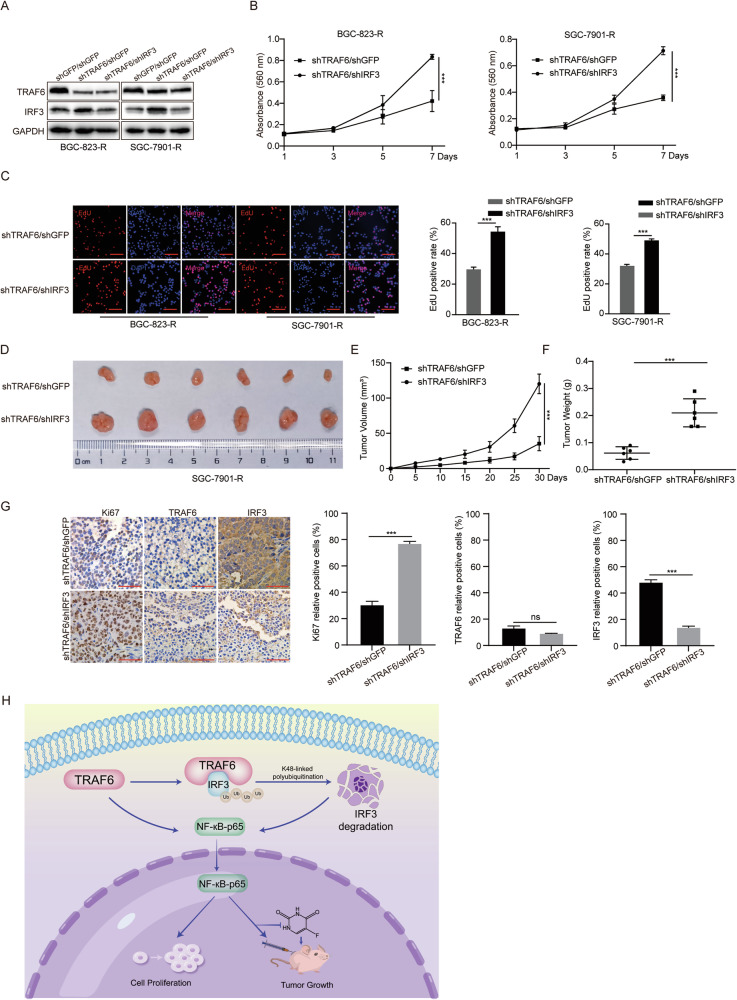
Depletion of IRF3 promotes the proliferation and tumor growth in 5-FU-resistant GC cells with TRAF6 knockdown. **A** Western blot analysis was performed after IRF3 knockdown in TRAF6-knockdown cells. **B** MTT assay was performed to examine the proliferation ability of BGC-823-R and SGC-7901-R cells. **C** EdU staining assay was performed to detect the amount of DNA synthesis in BGC-823-R and SGC-7901-R cells. Scale bar=50 μm. **D**–**F** Photographs, growth monitoring and weights of the indicated xenograft tumors. Scale bar = 1 cm, *n* = 6. **G** Representative IHC images of the positive expression levels of Ki67, TRAF6 and IRF3. Scale bar = 50 μm. Quantification of Ki67, TRAF6 and IRF3 expression. Data were expressed as the mean ± SD, *n* = 3. Student’s t-test was performed to analyze significance. *P < 0.05, **P < 0.01, ***P < 0.001. **H** A schematic representation of the potential mechanisms by which TRAF6 induces GC cells insensitivity to 5-FU by targeting IRF3.

Previously, we demonstrated an interaction between TRAF6 and IRF3, with an inverse regulatory relationship between them. Next, we aimed to determine if TRAF6-mediated proliferation of 5-FU-resistant GC cells and tumor growth were affected by IRF3. We knocked down IRF3 expression in 5-FU-resistant GC cells with stable TRAF6 knockdown (shTRAF6/shIRF3) to examine the expression of TRAF6 and IRF3 (Fig. 7A). MTT test, plate cloning and EdU assays determined proliferation-related indicators. MTT and EdU tests showed faster cell growth rate and higher EdU positivity in double knockdowns versus controls (Fig. 7B, C). Plate cloning experiments proved shTRAF6/shIRF3 restored cell cloning ability significantly (Supplementary Fig. 5A). The subcutaneous xenograft assays showed, the tumor growth rate, volume and weight of 5-FU-resistant GC cells were restored (Fig. 7D–F). IHC analysis showed that Ki67 positive rate was recovered after IRF3 downregulation in TRAF6 knockdown cells. IRF3 positive rate and control were comparable, but the TRAF6 positive rate was unaffected by IRF3 knockdown (Fig. 7G). All data showed IRF3 is a substrate protein of TRAF6 and its loss is critical for TRAF6-promoted proliferation of 5-FU-resistant GC cells.

Based on these findings, we summarized the TRAF6/IRF3 axis model regulating 5-FU-resistant GC cells (Fig. 7H): TRAF6 induces Lys70 ubiquitination of IRF3 by binding to IRF3-Δ (SR + IAD) (1-190aa), promotes the degradation of IRF3 protein, increases the NF-κB-p65 nuclear translocation, and ultimately promotes the proliferation and tumorigenesis of 5-FU-resistant GC cells.

## Discussion

Although the morbidity and mortality of gastric cancer have steadily declined over past half century [24, 25], the incidence in East Asia remains high, accounting for 32.7% of global cases. As a first-line drug for clinical treatment of GC, 5-FU has significant inhibitory effects. However, resistance to 5-FU leads to poor chemotherapy outcomes, with most patients worldwide currently facing the thorny problem of poor prognosis [26–28]. In this study, we demonstrated that targeting TRAF6 can significantly reduce 5-FU resistance in GC.

Firstly, through proteomic sequencing analysis of 5-FU-resistant GC cells and 5-FU-sensitive GC cells, we found that TRAF6 was highly expressed in 5-FU-resistant GC cells. Analysis of the GEPIA and KM plotter databases showed its high expression correlated with poor patient prognosis, consistent with the previous reports that TRAF6 overexpression is associated with progression of many cancers [29–31]. A 2015 study reported that 5-FU chemoresistance was promoted through low Smad4 expression which activate PI3K/Akt/CDC2/survivin cascade and reduce cell cycle arrest in colorectal cancer cells [32]. We found that TRAF6 depletion blocked the cell cycle in G2/M phase, as evidenced by flow cytometry and related cyclin expression analysis. Subsequent in vitro and in vivo experiments, including subcutaneous tumor formation in mice, demonstrated the biological roles of TRAF6 in development of 5-FU resistance in GC cells.

TRAF6 is involved in signal transduction in multiple signaling pathways, including the typical TRAF6 signaling mechanism, namely TRAF6-TAK1-MAPK/IKK-NF-κB. TRAF6 can also activate PI3K and downstream AKT/PKB signaling pathways. Additionally, TRAF6 can promote Lys-63 linked ubiquitination of STAT3 without changing the expression of STAT3 protein, further activating MCL1 and Bcl-2 [33, 34]. In the Notch signaling pathway, TRAF6 activates the TGFβ receptor facilitating recruitment of PS1 to the TGFβRI complex and promoting the cleavage of TGFβRI into the nucleus. PS1 can also promote the cleavage of the intracellular segment of the Notch receptor into the nucleus [35]. Nuclear factor-κB (NF-κB) plays a critical role in the NF-κB signaling pathway. This pathway regulates many physiological processes and playing a pivotal role in the tumorigenesis, progression, metastasis and treatment resistance in human tumors [36, 37]. Studies have shown that TRAF6 directly binds to the intracellular domain of receptors, such as CD40, activating NF-κB [38]. TRAF6 has been shown to activate NF-κB via the TRAF6-TAB2 axis in tumors, promoting lung cancer progression [39]. We found upregulated TRAF6 in 5-FU-resistant GC cells promoted the nuclear translocation of NF-κB-p65, while TRAF6 downregulation inhibited this process. In fact, as a type I interferon regulatory factor, IRF3 is traditionally known to function through transcriptional activity, and related to antiviral responses. Pattern recognition receptors activate IRF3 and NF-κB during viral infections [40], initiating downstream signals to interfere with viral life cycle. In recent years, researchers have begun to focus on its non-transcriptional role, such as interaction with other proteins or regulation of cellular signaling pathways. However, the role of IRF3 and NF-κB in the context of tumor cell resistance to chemotherapy remain elusive. In this study, based on the discovery of an interaction between TRAF6 and IRF3, we further expanded their contribution to the nuclear translocation of NF-κB-p65, that is, the TRAF6-IRF3 axis induced the activation of NF-κB-p65 and finally promoted chemoresistance of GC cells to 5-FU.

GC cells exposed to chemotherapeutic drugs can develop various resistance mechanisms, such as regulating glucose metabolism, oxidative stress response, mitochondrial activity, and EMT, enabling their survival against anticancer drugs. Studies have shown that targeting markers, related pathways, or microenvironmental niches of cancer stem cells (CSCs) may impair chemotherapy resistance [41, 42]. CD44 is the earliest marker of CSCs in GC [43]. Targeting ZFP64 can enhance the therapeutic effect in GC patients who are resistant to albumin-bound paclitaxel [44]. Additionally, circCPM can affect autophagy and 5-FU resistance in GC, which is achieved by targeting PRKAA2 [45]. In recent years, protein post-translational modification has been recognized as an important regulatory mechanism of tumor chemoresistance. Protein ubiquitination, one of these modifications, play a pivotal role in in various cellular processes such as protein localization, degradation, cell cycle proliferation and cell signal transduction. Many functions of the ubiquitin system are realized through mono-ubiquitin or polyubiquitin chain formation on target substrates. Ubiquitin comprises 76 amino acids with seven lysine residues [46]. These residues can undergo ubiquitination to form unique polyubiquitin chains. The four major E3 ubiquitin ligases include HECT type, U-box type, RING-finger type and RBR type. Among these, HECT type and RING-finger type have been clearly demonstrated to participate in the regulation of cancer progression [47–49]. Monomeric RING E3 ligases in the RING-finger type possess domains for substrate binding and ubiquitination, and also harbor self-ubiquitination functions, exemplified by COP1, Mdm2, and TRAF6 [50]. The K48/K63 ubiquitination of TRAF6 was reported to regulate protein interaction, consistent with our findings on the TRAF6-IRF3 interaction. Importantly, we found that TRAF6 promotes the degradation of IRF3 through K48-mediated ubiquitin. Previous studies have reported that ubiquitination at Lys193 and Lys313 on IRF3 mediates apoptosis via activation of RLR pathway [17]. Similarly, MIDI has been shown to induce multi-ubiquitination of K48 at Lys313 to promote IRF3 degradation [23]. However, we demonstrate that IRF3 ubiquitin substrate site is Lys70 in GC chemoresistant cells. Moreover, IRF3 depletion promotes the proliferation of 5-FU-resistant GC cells with TRAF6 knockdown. Therefore, GC patients with high TRAF6 expression and 5-FU resistance might benefit from combined treatment with 5-FU and IRF3 agonists, which warrants further investigation.

Overall, our work reveals the important role of TRAF6 in the proliferation of 5-FU-resistant GC cells. TRAF6 interacted with IRF3-Δ (SR + IAD) (1-190aa), and promotes NF-κB-p65 translocation to the nucleus by facilitating the ubiquitin mediated degradation of IRF3, resulting in 5-FU resistance. Therefore, therapeutically targeting TRAF6 represents a promising strategy for cancer treatment and overcoming drug resistance.

## Supplementary information

Original western blot

Supplementary File

## Data Availability

All data and materials in this paper are provided in the main text and supplementary materials.
