# Supplementary material for: Targeting TRAF6/IRF3 axis to inhibit NF-κB-p65 nuclear translocation enhances the chemosensitivity of 5-FU and reverses the proliferation of gastric cancer
Source: Cell Death Dis. 2024 Dec 20;15(12):924. doi: 10.1038/s41419-024-07290-5 (PMC11662071; doi:10.1038/s41419-024-07290-5)
Supplement: Supplementary file 2 — Supplementary File [file 41419_2024_7290_MOESM2_ESM.doc]

**Supplementary Table. 1 Antibodies used in the experiment**

| Antibody | | Company | Product Code |
| --- | --- | --- | --- |
| TRAF6 | Cell Signaling Technology | | 67591 |
| Ki67 | Cell Signaling Technology | | 9449 |
| IRF3 | Proteintech | | 66670-1-Ig |
| MYC-tag (Mouse) | Proteintech | | 60003-2-Ig |
| MYC-tag (Rabbit) | Proteintech | | 16286-1-AP |
| Flag-tag (Mouse) | Proteintech | | 66008-4-Ig |
| NF-κB-p65 | Proteintech | | 80979-1-RR |
| HA-Tag | Proteintech | | 81290-1-RR |
| GAPDH  CDK1  Cyclin A2  Histone H3 | Proteintech  Proteintech  Proteintech  Proteintech | | 60004-1-Ig  19532-1-AP  18202-1-AP  17168-1-AP |

**Supplementary Table. 2 Sequences for shRNA.**

| Gene Name | Sequence | |
| --- | --- | --- |
| shTRAF6#1 | | CGGAATTTCCAGGAAACTATT |
| shTRAF6#2 | | CCTGGATTCTACACTGGCAAA |
| shIRF3 | | GCAGGAGGATTTCGGAATCTT |

**Supplementary Table. 3 qRT-PCR primer sequences.**

| Gene Name | Sequence (5’-3’) | |
| --- | --- | --- |
| TRAF6-forward | | CTTTCCAGCGACCCACAATCCC |
| TRAF6-reverse | | CTCCGAAGGCTACCCATGTCAAAG |
| GAPDH-forward | | ACGGATTTGGTCGTATTGGG |
| GAPDH-reverse | | CGCTCCTGGAAGATGGTGAT |

**Supplementary Figure Legends**

**
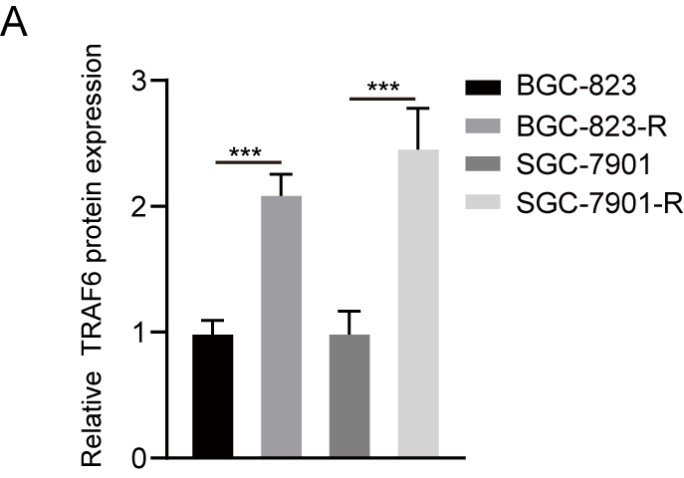
**

**Supplementary Figure. 1 High expression of TRAF6 enhances 5-FU resistance in GC cells.**

(A) The relative expression of TRAF6 protein was statistically analyzed.


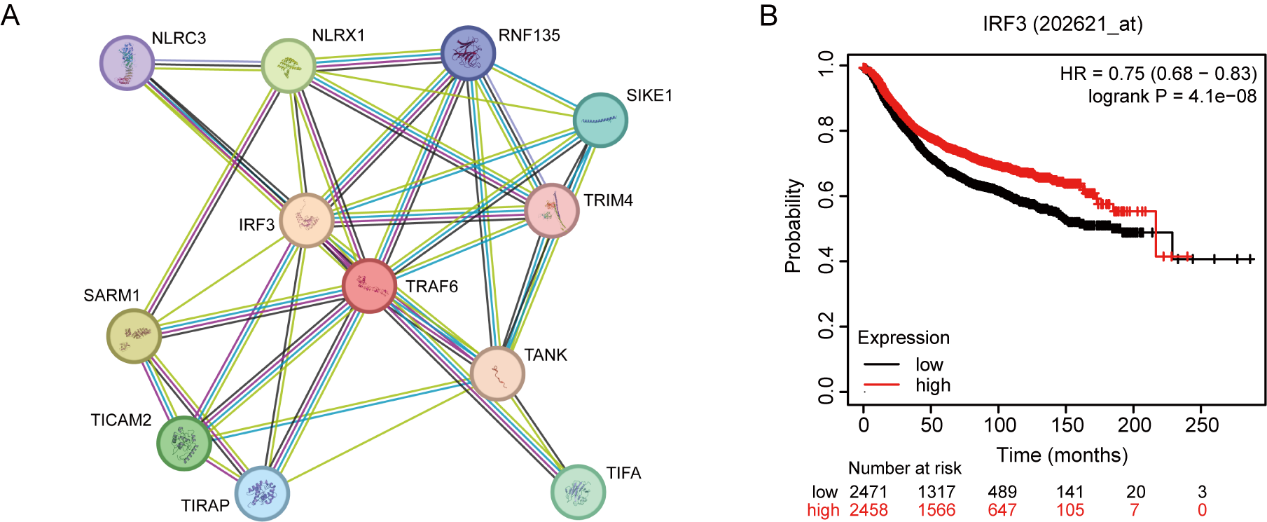


**Supplementary Figure. 2 TRAF6 interacts with IRF3**

(A) STRING database predicted the relationship between TRAF6 and IRF3 proteins. (B) Kaplan–Meier analysis of overall survival probability was performed using the Kaplan-Meier Plotter database.


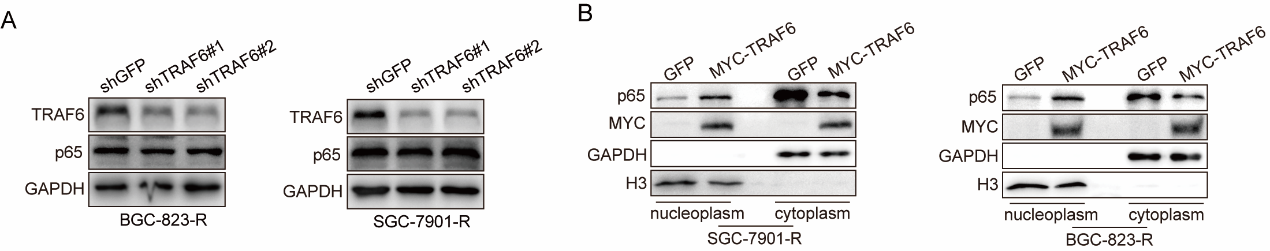


**Supplementary Figure. 3 Overexpression of TRAF6 promotes** **NF-κB-p65 nuclear translocation**

(A) Expression of endogenous p65 protein in TRAF6-knockdown BGC-823-R and SGC-7901-R cells was detected by Western blot. (B) Western blot was performed to detect the expression level of p65 in TRAF6-overexpressing cells.


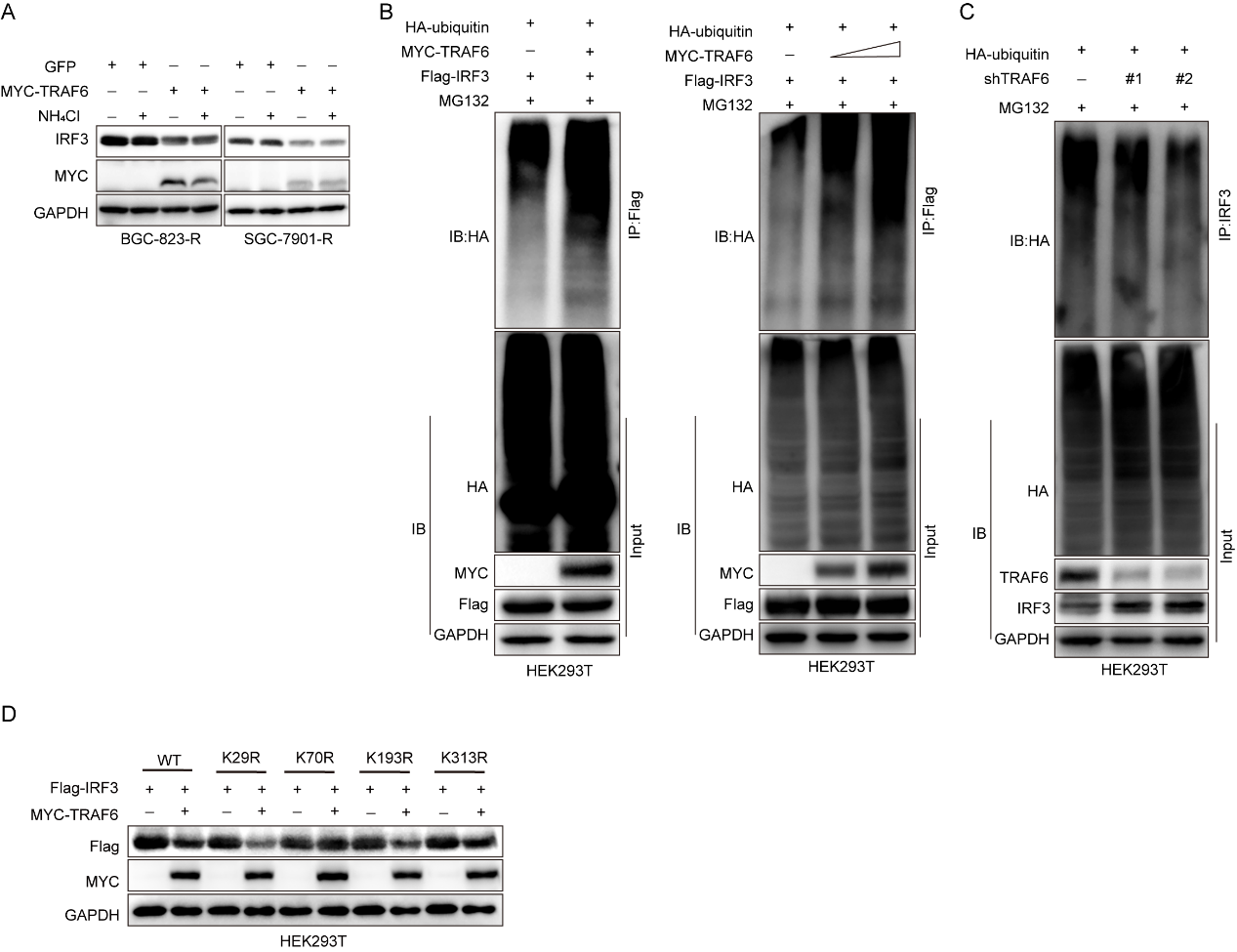


**Supplementary Figure. 4 TRAF6 stabilizes IRF3 through ubiquitination**

(A) Western blot analysis of control and TRAF6-overexpressing BGC-823-R and SGC-7901-R cells treated with NH_4_Cl (10 μM) for 6 h. (B, C) In the presence of MG132, with MYC-TRAF6 or shTRAF6, Flag-IRF3, and HA-UB plasmids were co-transfected into 293T cells for ubiquitination assays. (D) With or without MYC-TRAF6 and Flag-IRF3 (wild-type and single-point mutants K29R, K70R, K193R, K313R) were co-transfected into 293T cells, Western blot was performed to detect the expression level of Flag-IRF3 and its mutants.


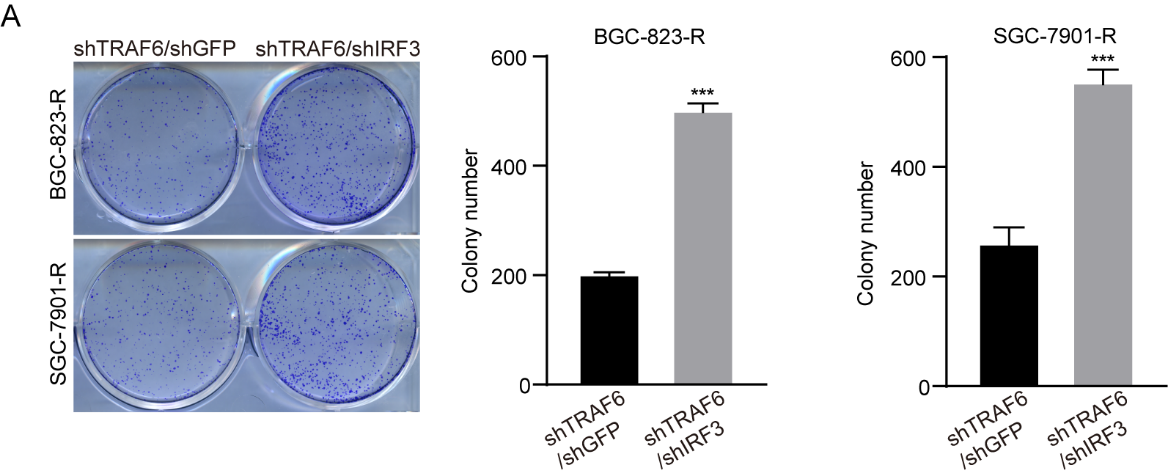


**Supplementary Figure. 5 Depletion of IRF3 restores the cloning ability of 5-FU-resistant GC cells**

(A) Plate cloning assay was performed after IRF3 knockdown in TRAF6-knockdown cells.
